# Antifungal Properties of Nerolidol-Containing Liposomes in Association with Fluconazole

**DOI:** 10.3390/membranes10090194

**Published:** 2020-08-20

**Authors:** Camila Fonseca Bezerra, José Geraldo de Alencar Júnior, Rosilaine de Lima Honorato, Antonia Thassya Lucas dos Santos, Josefa Carolaine Pereira da Silva, Taís Gusmão da Silva, Antonio Linkoln Alves Borges Leal, Thiago Sampaio de Freitas, Thiago Adler Tavares Vieira, Janaína Esmeraldo Rocha, Débora Lima Sales, José Maria Barbosa Filho, Gabriela Ribeiro de Sousa, Allyson Pontes Pinheiro, Jaime Ribeiro-Filho, Henrique Douglas Melo Coutinho, Maria Flaviana Bezerra Morais-Braga, Teresinha Gonçalves da Silva

**Affiliations:** 1 Department of Pharmaceutical Sciences, Federal University of Pernambuco-UFPE, Recife-PE 50670-901, Brazil; camilawasidi@gmail.com; 2 Department of Pharmacy, Federal University of Ceará-UFC, Fortaleza-CE 60020-181, Brazil; junioralencar727@gmail.com; 3 Department of Biological Sciences, Regional University of Cariri-URCA, Crato-CE 63105-010, Brazil; rosilainehonorato@gmail.com (R.d.L.H.); thassyalucas@hotmail.com (A.T.L.d.S.); carol.bio1881@outlook.com (J.C.P.d.S.); taisgusmao96@gmail.com (T.G.d.S.); allysson.ponheiro@urca.br (A.P.P.); flavianamoraisb@yahoo.com.br (M.F.B.M.-B.); 4 Department of Biological Chemistry, Regional University of Cariri-URCA, Crato-CE 63105-010, Brazil; antoniolinkoln@hotmail.com (A.L.A.B.L.); thiagocrato@hotmail.com (T.S.d.F.); thiago.a.t.vieira@gmail.com (T.A.T.V.); janainaesmeraldo@gmail.com (J.E.R.); debora.lima.sales@gmail.com (D.L.S.); 5 Department of Pharmacy, Federal University of Paraíba-UFPB, João Pessoa-PB 58051-900, Brazil; jbarbosa@ltf.ufpb.br (J.M.B.F.); grsousafarm@gmail.com (G.R.d.S.); 6 Gonçalo Moniz Institute, Oswaldo Cruz Foundation, Salvador-BA 40296-710, Brazil; jaimeribeirofilho@gmail.com; 7 Departament of Antibiotics, Federal University of Pernambuco-UFPE, Recife-PE 50670-901, Brazil; teresinha100@gmail.com

**Keywords:** nerolidol, liposomes, fluconazole, *candida* dimorphism, antifungal resistance inhibition

## Abstract

(1) Background: Infections by *Candida* species represent a serious threat to the health of immunocompromised individuals. Evidence has indicated that nerolidol has significant antifungal properties. Nonetheless, its use is restricted due to a low water solubility and high photosensitivity. The incorporation into liposomes may represent an efficient alternative to improve the physicochemical and biopharmaceutical properties of this compound. The present study aimed to characterize the antifungal properties of liposomal nerolidol, alone or in combination with fluconazole. Of note, this is the first study reporting the antifungal activity of liposomal nerolidol and its potentiating effect in association with fluconazole. (2) Methods: The Inhibitory Concentration 50%-IC_50_ and minimum fungicide concentrations (MFC) of the substances against *Candida albicans* (CA), *Candida tropicalis* (CT), and *Candida krusei* (CK) were established by subculture in a solid medium. To evaluate the antifungal-enhancing effect, the MFC of fluconazole was determined in the presence or absence of subinhibitory concentrations of nerolidol (free or liposomal). The analysis of fungal dimorphism was performed through optical microscopy and the characterization of liposomes was carried out considering the vesicular size, polydispersion index, and zeta medium potential, in addition to a scanning electron microscopy analysis. (3) Results: The physicochemical characterization revealed that liposomes were obtained as homogenous populations of spherical vesicles. The data obtained in the present study indicate that nerolidol acts as an antifungal agent against *Candida albicans* and *Candida tropicalis*, in addition to potentiating (only in the liposomal form) the effect of fluconazole. However, the compound had little inhibitory effect on fungal dimorphism. (4) Conclusions: The incorporation of nerolidol into liposomes improved its antifungal-modulating properties.

## 1. Introduction

Infections by *Candida* species represent a serious threat to the health of immunocompromised individuals, including transplant patients, cancer patients, and those undergoing immunosuppressive drug therapy [1,2]. These microorganisms are the most frequent causative agents of invasive fungal infections, being commonly isolated from intensive care unit (ICU) patients [3]. Accordingly, *Candida* infections represent about 98% of the cases of fungemia associated with prolonged neutropenia in cancer patients [4,5,6].

The pharmacological treatment with azole antifungals is the mainstay of antifungal therapy in patients with hematological malignancies, autoimmune diseases, and those undergoing solid organ or stem cell transplantation [7]. However, the prolonged use (above 4 weeks) of these drugs has been associated with high treatment costs, the development of resistance, and significant toxicity [8], justifying the search for novel therapeutic agents to treat invasive fungal infections in immunocompromised patients.

Consistent evidence has demonstrated that aromatic alcohols such as tryptophol, tyrosol, phenylethyl alcohol, dodecanol, farnesic acid, trans-farnesol, and trans-nerolidol function as self-regulatory molecules in quorum sensing mechanisms associated with fungal differentiation, i.e., from yeast to hyphae. Since dimorphism has a significant impact on the pathogenesis of *Candida* species, the investigation of compounds capable of inhibiting this phenomenon may represent a promising strategy for the development of new antifungal drugs [9,10,11].

Nerolidol, a sesquiterpene alcohol found to be a component of many essential oils, has been widely used as a flavoring agent in the food industry [12]. Importantly, increasing evidence has identified nerolidol as a bioactive compound with antibacterial [13], antifungal [14,15], anti-inflammatory [16] and antitumor [17,18] properties. However, despite these promising pharmacological activities, the therapeutic use of nerolidol formulations is restricted due to a low water solubility and high photosensitivity [13,19]. 

In this context, our group has recently demonstrated that the incorporation of nerolidol into carrier nanoparticles, including liposomes, improved its physicochemical characteristics, which could result in improved biopharmaceutical properties [20]. Accordingly, studies have shown that liposomes can increase absorption, improve distribution, and prolong biological half-lives, thus improving the therapeutic index of several drugs [21,22].

Thus, it is hypothesized that the incorporation of nerolidol into liposomes will result in the improved anti-*Candida* activity of nerolidol alone or associated with fluconazole. Therefore, the present study aimed to evaluate the effect of these treatments on the growth and dimorphism of different *Candida* strains. Of note, this is the first study reporting the antifungal activity of liposomal nerolidol and its antifungal-enhancing effects in association with fluconazole.

## 2. Experimental Section

### 2.1. Preparation of Liposomes 

The lipids used in the preparation of liposomes (purity > 99%) were purchased from Sigma Aldrich Co. (St. Louis, MO, USA). Each liposome batch was prepared by weighing 5 mg of dipalmitoylphosphatidylcholine (DPPC), 3 mg of dipalmitoylphosphatidylserine (DPPS), and 1 mg of cholesterol (CHOL) and 1 mg (1 μL) of nerolidol. These substances were mixed and dissolved with 1 mL of a chloroform/methanol (purity > 99%, Dynamic Reagents-Diadema, SP, Brazil) solution (1:1). Nitrogen (White Martins, Rio de Janeiro, RJ, Brazil) was used to evaporate the solvents, after which a thin layer was formed on the tube wall. The tube was kept in a desiccator overnight (18 h) and then, 1 mL of PBS buffer (pH 7.2) was added. The tube was then subjected to a shaker and water bath (at 57 °C) to resuspend the liposomes, forming an emulsion, which was then submitted to an extruder (LiposoFastTM, Avestin) with polycarbonate membranes with pores 200 nm in diameter. This step was repeated 40 times producing a population of uniformly sized liposomes. Liposomes with no nerolidol incorporated were used as controls [23].

### 2.2. Physicochemical Characterization of Liposomes

The physicochemical characterization of liposomes was carried out by evaluating the following parameters: vesicular size, morphology, polydispersity index, and average zeta potential. The particle size was assessed through a dynamic light scattering (DLS) analysis, using serial dilutions with Milli Q water (1:10, 1:20, 1:50 and 1:100) at 25 °C. The particle size distribution was obtained by the polydispersity index (PI). The average zeta potential of the particles was calculated at 25 °C, using the microelectrophoresis technique associated with laser Doppler anemometry, by submitting the sample to an electric field. The analyses were performed using Zetasizer Nano ZS equipment (Malvern, version 6.20). The shape and morphology of the liposomes were analyzed by a scanning electron microscope (FEG Quanta 450 EDS/EBSD).

### 2.3. Analysis of Antifungal Activity

#### 2.3.1. Strains and Culture Media

Standard strains of *Candida* (CA INCQS 40006 (*Candida albicans*), CT INCQS 40042 (*Candida tropicalis*), and CK INCQS 40095 (*Candida krusei*)) were obtained from the Institute for Quality Control in Health (INCQS, FIOCRUZ, RJ) and incubated in Sabouraud Dextrose Agar (SDA, KASVI) at 37 °C for 24 h. Then, a sample of each colony was transferred to test tubes containing 3 mL of sterile saline, and the concentration was determined using a value of 0.5 on the McFarland scale [24]. Double-concentrated Sabouraud dextrose broth (SDB, HIMEDIA) was used in the microdilution tests while depleted potato dextrose agar (PDA) added to bacteriological agar was used in the morphological analysis.

#### 2.3.2. Drugs

Nerolidol (98% purity; cis/trans mixture) was obtained from Sigma Aldrich (St. Louis, MO, USA) and dissolved in dimethyl sulfoxide (DMSO, Merck, Darmstadt, Germany). Fluconazole (Capsule, Prati Donaduzzi) was dissolved in distilled water. Each treatment solution was prepared as previously described [25].

#### 2.3.3. Analysis of the Cell Viability and Determination of Inhibitory Concentration 50%-IC_50_

The cultures and treatments were performed as described by Javadpour and collaborators [26]. The solutions were prepared in eppendorf tubes each containing 1 mL of solution, which was composed of 900 μL of culture medium (broth Sabouraud dextrose—BSD) and 100 μL of the fungal suspension with 10^6^ CFU (Colony Forming Units) according to the MacFarland scale. The plate was filled in the numerical direction by adding 100 μL of this solution to each well, and then a serial microdilution technique was performed with the 100 μL solution of nerolidol or fluconazole, to achieve concentrations ranging from 8192 to 8 μg/mL. Wells containing only the inoculum in the BSD medium were used as the growth control. The diluent controls, in which the inoculum was replaced with 0.9% sodium chloride and a sterile medium, were also used. All tests were performed in quadruplicate. The plates were incubated at 37 °C for 24 h, and then, the readings were performed at 630 nm using a spectrophotometer (Thermoplate^®^). The data were used to determine the cell viability and calculate the IC_50_ of each treatment, as previously described [27].

#### 2.3.4. Determination of the Minimum Fungicidal Concentration (MFC) 

The minimum fungicide concentration was determined as described in a study by Ernst et al. [28], with a few modifications, according to Morais-Braga et al. [27]. It consisted of adding a sterile rod to each well of the microdilution plate, homogenizing the medium contained in the cavity, then it was subcultured in a Petri dish containing Saboraud dextrose agar and with the aid of a guide card with the corresponding numbering to the wells of the microdilution plate–5 µL of the test solution (medium + inoculum + natural product) was transferred in each quadrant listed. The plates were incubated at 37 °C. After 24 h of incubation, the growth of the *Candida* colonies in the plates was analyzed. The minimum fungicidal concentration (MFC) was defined as the lowest concentration in which no growth of fungal colonies was observed.

#### 2.3.5. Evaluation of the Antifungal Enhancing Activity in Association with Fluconazole

After evaluating the isolated action of nerolidol and fluconazole, this study analyzed the ability of nerolidol to enhance the antifungal activity of fluconazole. To this end, the MFC of fluconazole was determined in the presence or absence of nerolidol at a concentration equivalent to its MFC/16. The antifungal enhancing activity in association with fluconazole was investigated using the method described by Coutinho et al. [29].

#### 2.3.6. Analysis of Candida Morphological Changes

To investigate the effects of the treatments of fungal morphology, the development of hyphae was analyzed by optical microscopy in chambers containing sterile slides for the observation of yeasts, as previously described [30,31]. To this end, the chambers were added with 3 mL of PDA medium depleted by dilution, in the presence of nerolidol at concentrations equivalent to its MFC/8 or MFC/16. Aliquots of the subcultures were removed from the Petri dishes to make two parallel streaks in the solid medium (PDA), which were later covered with a sterile coverslip. The chambers were then placed in the oven at 37 °C for 24 h, and the images were recorded under optical microscopy (AXIO IMAGER M2-3525001980, ZEISS, Germany). Each slide was photographed and the length of the filament extensions (hyphae and pseudohyphae) was determined using the Zen 2.0 software [32].

### 2.4. Statistical Analysis

The IC_50_ was calculated by a nonlinear regression and expressed as the arithmetic mean ± standard error of the mean. The statistical significance was determined using a one-way ANOVA with Tukey’s post hoc test. The fungal growth was expressed as the arithmetic mean ± standard deviation and the statistical significance as calculated using two-way ANOVA with Bonferroni’s post hoc test. All experiments were performed in quadruplicate and analyzed using Graphpad Prism version 5.0.

## 3. Results

### 3.1. In Vitro Antifungal Activity of Nerolidol Alone and Incorporated into Liposomes

**Figure 1:**
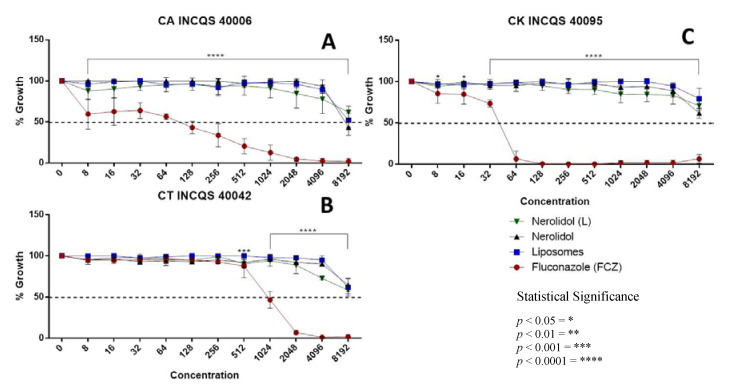
Growth curves of different *Candida* strains in the presence of fluconazole (FCZ), nerolidol and liposomal nerolidol (L). Liposomes without nerolidol were used as controls. (**A**) CA—*Candida albicans*; (**B**) CT—*Candida tropicalis*; (**C**) CK—*Candida krusei;* INCQS—National Institute for Quality Control in Health; concentration in µg/mL.

The growth curves of different *Candida* strains in the presence of nerolidol (free or liposomal) demonstrated that this compound presented a weak antifungal activity against all strains of *Candida* evaluated by this study, in contrast to the pharmacological control fluconazole (Figure 1). These findings can be observed through an analysis of the IC_50_ values shown in Table 1. While nerolidol demonstrated the strongest activity against *Candida albicans*, the incorporation of this compound into liposomes was found to present an enhanced antifungal activity only against *Candida tropicalis*, indicating a selective improvement of its antifungal action with regard to the type of strain.

### 3.2. Antifungal-Enhancing Activity of Liposomal Nerolidol in Association with Fluconazole

To evaluate the ability of nerolidol to modulate the antifungal activity of fluconazole, this work analyzed the growth curves of three different *Candida* strains in the presence of the standard antifungal drug alone, or in association with subinhibitory concentrations (MFC/16) of free or liposomal nerolidol. Of note, the MFC analysis found values above 16,384 μg/mL for all these treatments, confirming that they had little intrinsic antifungal activity.

**Figure 2:**
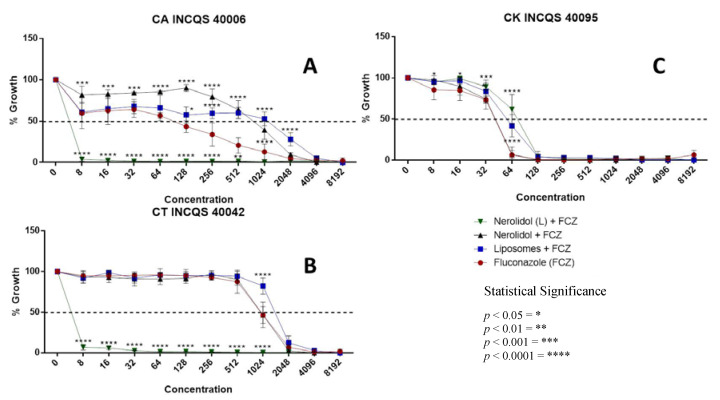
Growth curves of different *Candida* strains in the presence of fluconazole (FCZ) alone or in association with nerolidol, liposomal nerolidol (L) or liposomes. (**A**) CA—*Candida albicans*; (**B**) CT—*Candida tropicalis*; (**C**) CK—*Candida krusei;* INCQS—National Institute for Quality Control in Health.

The association with subinhibitory concentrations of liposomal nerolidol significantly increased the antifungal activity of fluconazole against *C. albicans* (Figure 2A) and *C. tropicalis* (Figure 2B), demonstrating a potentiating effect, which was not observed for the association of fluconazole with the unconjugated compound. It is worth mentioning that the association between liposomal nerolidol and fluconazole against *C. tropicalis* caused a 432-fold reduction in the MFC of fluconazole alone (Table 2). 

On the other hand, the simultaneous treatment with unconjugated nerolidol and fluconazole presented a significant action reducing effect against *C. albicans* and *C. krusei*, indicating an influence of the nanoformulation in the antifungal-modulating properties of the compound in association with the standard drug. Here, it is hypothesized that the controlled and constant release of the compound by the liposome can influence its action on the fungal membrane, inhibiting the growth of microorganisms.

### 3.3. Effects of the Treatments on Fungal Morphology

**Figure 3:**
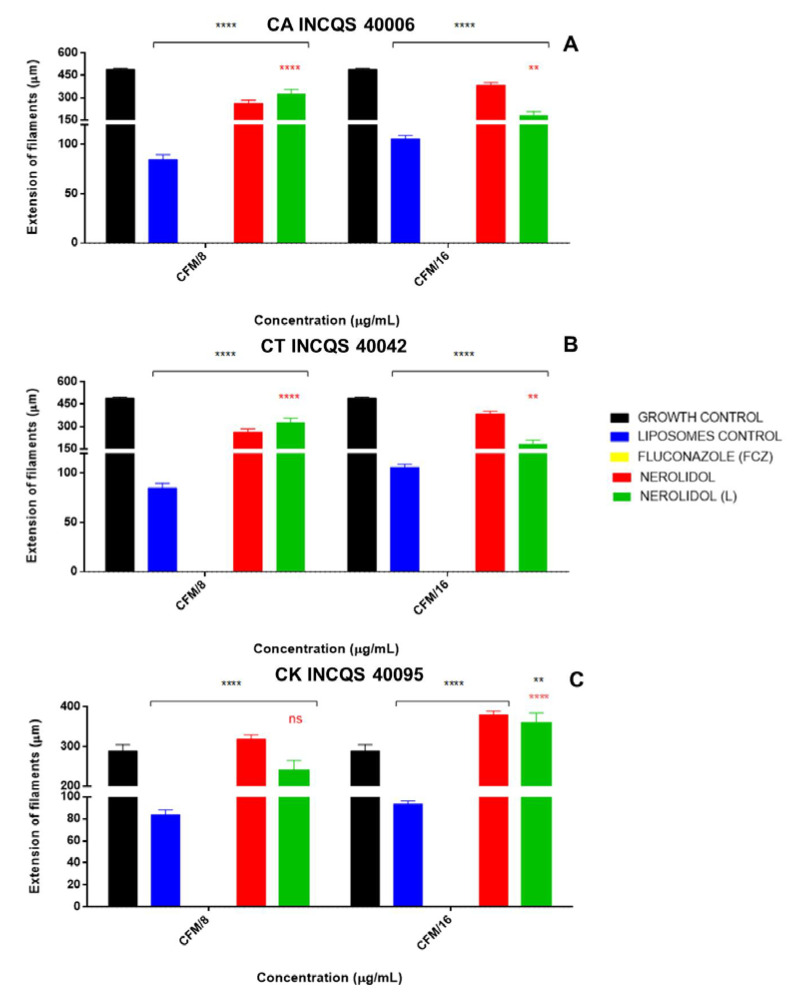
Effects of the in vitro treatment with fluconazole (FCZ), nerolidol and liposomal (L) nerolidol on the fungal morphology. Changes in the fungal morphology were expressed as a measure of the extension of filaments. Liposomes without nerolidol were used as controls. (**A**) CA—*Candida albicans*; (**B**) CT—*Candida tropicalis*; (**C**) CK—*Candida krusei*; INCQS—National Institute for Quality Control in Health. *p* < 0.01 = **, *p* < 0.0001 = ****.

Following the antifungal activity analysis, this study investigated the effects of the treatments on fungal dimorphism, one of the main virulence factors in *Candida* species. To this end, the compounds were used at concentrations equivalent to their MFC/8 (2048 μg/mL) and MFC/16 (1024 μg/mL). The in vitro treatment with fluconazole at both concentrations prevented the formation of filamentous structures, indicating that this drug is capable of inhibiting fungal dimorphism in *Candida albicans* (Figure 3A), *Candida tropicalis* (Figure 3B) and *Candida krusei* (Figure 3C). On the other hand, free or liposomal nerolidol had little inhibitory effect on dimorphism in *C. albicans* (Figure 3A) and *C. tropicalis*. In contrast, these treatments seemed to stimulate dimorphism in *Candida krusei*, as attested by the increase in the hyphae filaments compared with the untreated group (control).

**Figure 4:**
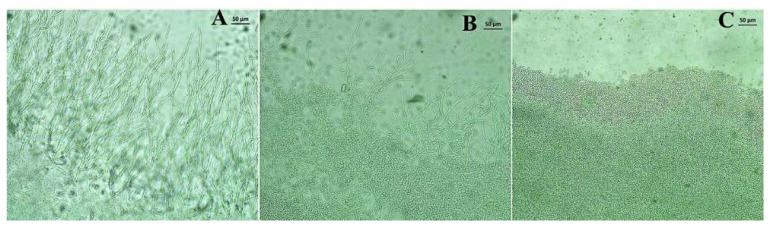
Optical microscopy images showing the effects of different treatments on the growth of fungal filaments. (**A**) Control; (**B**) nerolidol (CFM/8); (**C**) fluconazole CFM/8.

Figure 4 shows the optical microscopy images of the filaments in the presence of different treatments. The treatment with nerolidol (Figure 4B) caused a mild reduction in the length of the filaments, compared to the untreated group (Figure 4A), while treatment with fluconazole (Figure 4C) was shown to completely inhibit the growth of these structures.

### 3.4. Physicochemical Characterization of Liposomes

Liposomes were characterized under dynamic light scattering. Our analyses revealed that these nanoformulations were obtained as spherical and homogeneous populations of vesicles with satisfactory dimensions (Table 3). The control vesicles presented an average diameter of 185.46 nm, while the vesicles containing nerolidol presented an average diameter of 132.3 nm. These values correspond to the expected values for extrusions carried out using 200 nm membrane filters. The zeta potential evidenced loading surfaces with a residual negative charge, which indicates a significant stability to be used in different formulations.

These analyses were carried out with diluted samples using water as a dispersion medium, at 25 °C. Values are expressed as the means ± standard deviations

**Figure 5:**
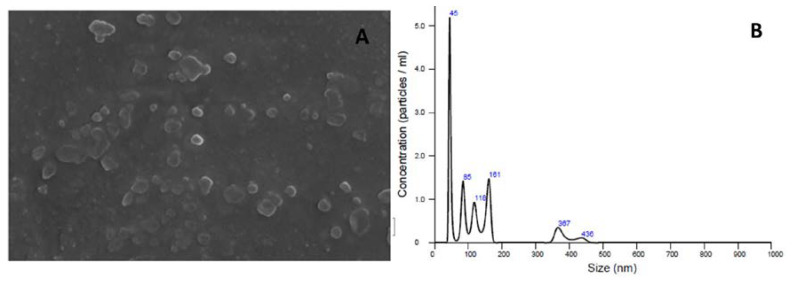
Scanning electron microscopy images (SEM) (**A**) and diagram of the distribution of the vesicles considering their size and concentration/mL (**B**).

The morphological analysis of the liposomes by scanning electron microscopy (SEM) (Figure 5A) revealed the presence of homogeneous populations of spherical vesicles with similar dimensions. Figure 5B shows the distribution of the vesicles considering their size and concentration/mL.

## 4. Discussion

The use of natural compounds in the treatment of fungal infections is considered a traditional alternative to the use of synthetic drugs [33]. On the other hand, increasing evidence has demonstrated that some natural products, in addition to having intrinsic pharmacological activities, can potentiate the activity of conventional drugs. In this context, in vitro studies have demonstrated the ability of nerolidol, a compound isolated from *Chamaecyparis obtusa*, to enhance the action of drugs such as nicardipine, hydrocortisone, carbamazepine, tamoxifen [34] and diclofenac [35].

The antifungal activity of nerolidol has been demonstrated by several studies, indicating that this compound has a potent action against *C. albicans* [36,37,38,39], and some evidence has suggested that this compound is more effective against susceptible strains. A study comparing the activity of a commercially acquired cis/trans-nerolidol mixture with that of a sample of the same compound isolated from the leaves demonstrated that the natural product showed a more potent antifungal activity against *C. albicans* [40]. Nevertheless, in the present study, nerolidol was found to present a weak antifungal activity and, except when incorporated into liposomes, showed no significant antifungal-enhancing activity in association with fluconazole. This drug was found to present excellent antifungal activity in all the tests carried out in the present research, proving its remarkable usefulness as a pharmacologic control. Fluconazole belongs to the most important class of antifungal drugs, the azoles, which have been widely used clinically, due to their notable safety and availability as oral and intravenous formulations [41].

After evaluating the antifungal and antifungal-modulating activities of both free and liposomal nerolidol, this study evaluated the ability of these treatments to inhibit fungal dimorphism, a crucial virulence phenomenon in *Candida* species. Our results indicate that nerolidol had little inhibitory effect on the growth of filamentous structures, indicating a weak inhibition of fungal dimorphism in *C. albicans* and *C. tropicalis*. However no significant effect was observed in the tests with *C. krusei*. A study by Martins and collaborators [9] demonstrated that the essential oil of *Piper claussenianum*, which had a high concentration of transnerolidol (81.4%), strongly inhibited the growth of hyphae. Potin et al. [42] evaluated the activity of nerolidol against *Sclerotium cepivorum* using the disk diffusion method (4 μg/disc). According to these authors, in addition to inhibiting the fungal growth, the compound caused morphological changes, inhibiting the formation of hyphae, possibly by causing damage to the integrity of the fungal membrane, corroborating the data found in the present study. 

As previously reported, a study analyzing the effect of a nerolidol-rich essential oil against *C. albicans* demonstrated a significant inhibition of fungal dimorphism, in addition to inhibiting biofilm formation by about 30 and 50% after incubation for 24 h and 48 h, respectively. The study also found that the combination of the essential oil with fluconazole resulted in significant synergistic effects. Vitali et al. [43] demonstrated that the essential oil of *Carpathian thymus*, which had nerolidol as a major component, presented significant antifungal activities both alone and associated with nystatin. Of note, this is to date, the first study reporting the antifungal properties of liposomal nerolidol.

The physicochemical characterization demonstrated that liposomes were obtained as homogeneous populations with little variation in size between the groups, which was confirmed by the scanning electron microscopy analysis. The average size of liposomal nerolidol was 132.3 nm, which is in accordance with the standard values reported in the literature [44,45,46]. Nevertheless, according to Azzi et al. [20], the variations in the size of liposomal vesicles may occur due to changes in the arrangement of lipids induced by the incorporated substance.

Despite having little intrinsic antifungal activity against *Candida* species, nerolidol was found to exhibit potent antifungal activity against *Microsporum gipseum* both in vitro and in vivo, with excellent results in the treatment of dermatophytosis [47]. These findings are corroborated by phytochemical studies demonstrating that this sesquiterpene was identified as a major compound in numerous plants with proven antimicrobial activity [18]. In this context, Cazella et al. [48] demonstrated that an essential oil obtained from *B. dracunculifolia* containing spathulenol (27.43%) and nerolidol (23.06%) as major components, showed significant antimicrobial activity against a wide variety of microorganisms. However, the mechanism of antifungal action of nerolidol remains to be better understood, although some evidence has suggested that this compound directly alters the cell membrane permeability, which can lead to cell death [49,50].

## 5. Conclusions

The data obtained in the present study indicate that nerolidol acts as an antifungal agent against *Candida albicans* and *Candida tropicalis*, in addition to potentiating (only in the liposomal form) the effect of fluconazole. However, the compound had little inhibitory effect on fungal dimorphism and, therefore, further studies are needed to characterize the antifungal properties of nerolidol incorporated into liposomes and other carrier nanoparticles, as well as investigate their potential applications in combating antifungal resistance.

## Figures and Tables

**Table 1:** Inhibitory Concentration 50%-IC_50_ values (µg/mL) of nerolidol against *Candida* strains.

Substance	CA INCQS 40006 IC_50_ (µg/mL)	CT INCQS 40042 IC_50_ (µg/mL)	CK INCQS 40095 IC_50_ (µg/mL)
Nerolidol	1000.23 ± 1000.0	25,000.29 ± 5000.53	15,400.1 ± 1051.39
Nerolidol (L)	16,000.41 ± 2000.8	12,800.27 ± 1112.32	47,000.67 ± 12,000.52
Fluconazole	55.98 ± 12.11	1000.99 ± 118.25	35.68 ± 1.74
Liposome	13,000.00 ± 1000.41	23,491.13 ± 823.49	40,000.75 ± 7000.1

Legend: CA—Candida albicans; CT—Candida tropicalis; CK—Candida krusei. L—liposomal.

**Table 2:** IC_50_ values (µg/mL) of fluconazole associated with nerolidol against *Candida* strains.

Treatment	CA INCQS 40006 IC_50_ (µg/mL)	CT INCQS 40042 IC_50_ (µg/mL)	CK INCQS 40095 IC_50_ (µg/mL)
Nerolidol + FCZ	800.86 ± 83.64	1000.72 ± 11,303	41.93 ± 4.85
Nerolidol (L) + FCZ	2.56 ± 0.03	2.70 ± 0.06	72.69 ± 5.62
Fluconazole	55.98 ± 12.11	1000.99 ± 118.25	35.68 ± 1.74
Lipossome + FCZ	788.10 ± 142.15	1800.11 ± 164.51	61.33 ± 6.75

Legend: CA—Candida albicans; CT—Candida tropicalis; CK—Candida krusei. L—liposome; FCZ—fluconazole.

**Table 3:** Vesicular size (VS), polydispersity index (PI), and zeta potential (ZP) of liposomes.

Formulation	Size (nm)	PI	ZP (mV)
Control liposome	185.46 ± 3.76	0.48 ± 0.01	−40.9 ± 0.96
Liposomal nerolidol	132.3 ± 108.3	0.42 ±0.02	−42.6 ± 0.91
